# Functional tuning of Vascular L-type Ca^2+^ channels

**DOI:** 10.3389/fphys.2022.1058744

**Published:** 2022-11-15

**Authors:** Galina Yu Mironova, Nadia Haghbin, Donald G. Welsh

**Affiliations:** Robarts Research Institute, Department of Physiology and Pharmacology Schulich School of Medicine, The University of Western Ontario, London, ON, Canada

**Keywords:** vascular smooth muscle, cooperative gating, functional coupling, L-type Ca2+ channels, Ca2+ sensitization, channel trafficking

## Abstract

Vascular smooth muscle contraction is intimately tied to membrane potential and the rise in intracellular Ca^2+^ enabled by the opening of L-type Ca^2+^ channels. While voltage is often viewed as the single critical factor gating these channels, research is starting to reveal a more intricate scenario whereby their function is markedly tuned. This emerging concept will be the focus of this three-part review, the first part articulating the mechanistic foundation of contractile development in vascular smooth muscle. Part two will extend this foundational knowledge, introducing readers to functional coupling and how neighboring L-type Ca^2+^ channels work cooperatively through signaling protein complexes, to facilitate their open probability. The final aspect of this review will discuss the impact of L-type Ca^2+^ channel trafficking, a process tied to cytoskeleton dynamics. Cumulatively, this brief manuscript provides new insight into how voltage, along with channel cooperativity and number, work in concert to tune Ca^2+^ responses and smooth muscle contraction.

## Introduction

### Background: Foundational basis of smooth muscle contraction

**FIGURE 1:**
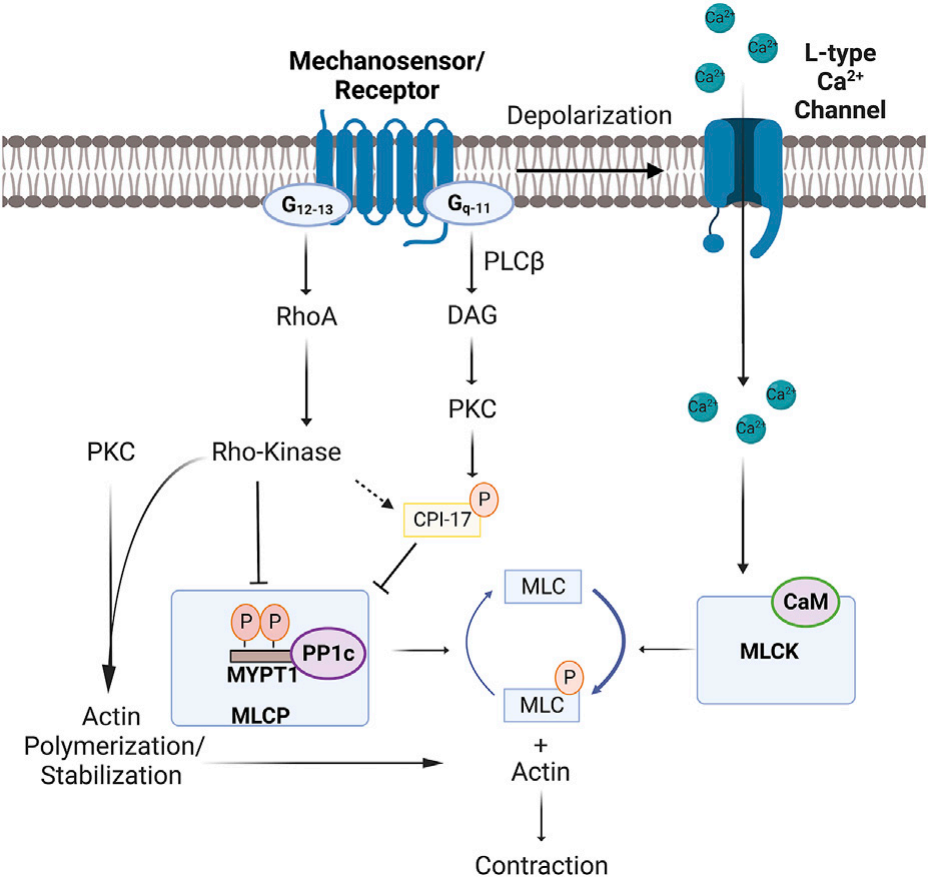
Mechanisms of cross-bridge cycling and MLC20 phosphorylation in vascular smooth muscle. Mechanosensor/receptor activation induces depolarization by activating transient receptor potential channels and inhibiting hyperpolarizing K+ currents. Depolarization opens L-type Ca2+ channels, with the resulting influx of Ca2+ enhancing MLCK and cross-bridge cycling through binding to CaM. Conversely, G-protein coupled receptors further enhance contraction by inhibiting MLCP (aka-Ca2+ sensitization) through two regulatory pathways. The first entails PKC activation by PLC-β and DAG, a series/threonine kinase that phosphorylates: 1) CPI-17 to inhibit PP1c; or 2) target proteins that set actin stabilization/polymerization. The second involves G12-13 activation of RhoA and Rho-kinase, the latter of which controls the phosphorylation state of: 1) MYPT1 (Threonine–696 and Threonine–853 (mouse numberings)); or 2) target proteins that set actin polymerization. Note, Ca2+ release from the sarcoplasmic reticulum, resulting from receptor-induced IP3 production, can also contribute to MLCK activation. Abbreviations: MLCK, Myosin light chain kinase; MLCP, Myosin light chain phosphatase, PLC, Phospholipase C; DAG, Diacylglycerol; CPI-17, Cytosolic phosphatase inhibitory protein of 17-kDa; IP3, Inositol trisphosphate; MYPT1, Myosin light chain phosphatase targeting subunit 1; MLC, Myosin regulatory light chain; CaM, Calmodulin; PKC, Protein Kinase C; PP1c, Type 1 protein phosphatase 1c.

Mechanical and chemical stimuli initiate vascular smooth muscle contraction through transduction pathways that enhance myosin light chain phosphorylation (Davis et al., 1999). This key biological event is set by the balance of two central enzymes, those being myosin light chain kinase and myosin light chain phosphatase (Takashima, 2009). Myosin light chain kinase is a serine/threonine-specific protein kinase responsible for phosphorylating Ser19 on the N-terminus of the regulatory light chain of the motor protein myosin-II (Figure 1). This enzyme’s activity is intimately tied to intracellular Ca^2+^ ([Ca^2+^]_i_) and its binding to low-affinity sites of kinase-bound Calmodulin, a messenger protein that interferes with the autoinhibitory domain (Herring et al., 2006; Schaub, 2007; Walsh, 2011). In contrast, myosin light chain phosphatase is a holoenzyme composed of three subunits: a 38 kDa catalytic subunit of type 1 protein phosphatase (PP1c), a 110–130 kDa regulatory subunit (MYPT1), and a small 20 kDa subunit of unknown function (Ding et al., 2006). MYPT1 is a key regulator of activity as phosphorylation at Thr853 or Thr696 inhibits PP1c, pushing the kinase-phosphatase balance towards enhanced myosin light chain phosphorylation and smooth muscle contraction. The control of MYTP1 phosphorylation is, in turn, set by signal transduction pathways tied to G-protein receptors, two of note being G_q/11_ and G_12/13_. The RhoA/Rho-kinase pathway is particularly important, and when inhibited pharmacologically (e.g., Y27632 or H1152), MYPT1 phosphorylation, myosin light chain phosphatase activity and smooth muscle contraction are diminished (Fu et al., 1998; Anneloes Martinsen et al., 2012). Downstream signaling proteins such as PKC also limit myosin light chain phosphatase activity by phosphorylating CPI-17, a direct inhibitor of the catalytic unit PP1c (Deng et al., 2002).

Ca^2+^ sensitization is a colloquial term used in vascular biology to describe how force development can be tuned relative to the rise in [Ca^2+^]_i_, the latter set by membrane depolarization. While this term is traditionally tied to phosphatase modulation, its mechanistic underpinnings have expanded to regulatory processes linked to actin-myosin interaction and the structures responsible for force transmission. As to the former, consider proteins such as Caldesmon, which, when bound to actin, stabilize it and impairs myosin ATPase activity (Clark et al., 1986). G-protein-linked signaling proteins like PKC diminish Caldesmon’s inhibitory effects by loosening its physical binding to the thin filaments (Clark et al., 1986). Calponin is another example of an actin-myosin binding protein inhibiting ATPase activity through an interaction with and phosphorylation by RhoA/Rho-kinase (Kaneko et al., 2000). With respect to the latter, consider current experimental interest in cytoskeletal remodeling, an event strongly, although not exclusively, tied to the state of actin polymerization. This dynamic process is regulated by several transduction pathways, one of note to G-protein coupled receptors and downstream Rho/Rho-kinase signaling being LIM kinase regulation of Cofilin, a protein that guides actin depolymerization (Walsh et al., 2013).

The synopsis above briefly highlights how vascular smooth muscle, through multiple points of regulation, can tune contractile development to a voltage-dependent rise in [Ca^2+^]_i_. Decidedly absent from this discussion is whether L-type Ca^2+^ channels themselves can be functionally tuned. This idea was first raised by Fallon and colleagues who noted that these channel’s C-termini interact with one another (Fallon et al., 2009), facilitating a state whereby the opening of one channel fosters the opening of a companion. The result of said “functional coupling” would be enhanced Ca^2+^ influx and contraction at a defined voltage (Dixon et al., 2022). An alternative means of so called “tuning” would be to traffic additional L-type Ca^2+^ channels to the plasma membrane to enhance cluster formation and cooperative gating (Ghosh et al., 2018). Both aspects of regulatory control (i.e., Cooperative gating and Channel trafficking) will be discussed in the subsequent chapters by highlighting key literature and classic experiments.

## Cooperative gating of L-type Ca2+ channels

**FIGURE 2:**
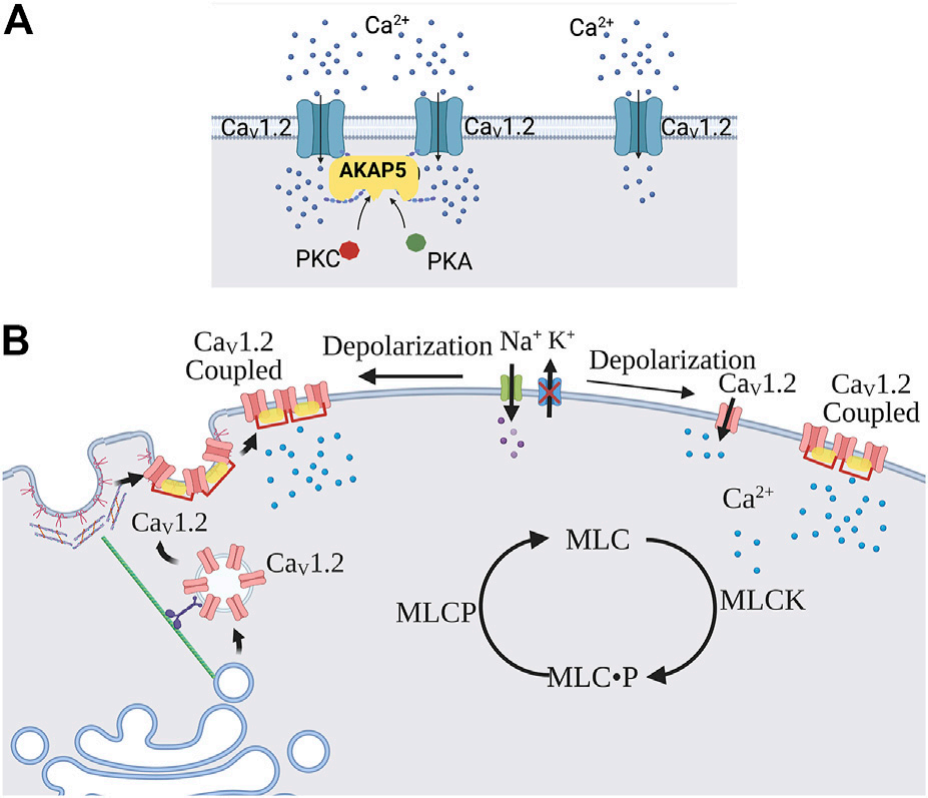
Cooperative gating and trafficking of L-type Ca^2+^ channels. **(A)** L-type Ca^2+^ channels can work as independent channels or in a cooperative gating manner due to proximate localization and the formation of connections at the Ca_V_1.2 C-terminus. Cooperatively gated channels exhibit functional coupling properties, such as synchronous opening, higher Po, and more persistent current compared to individually gated channels. AKAP5 (mouse numbering), PKC, and PKA play a critical role in the promotion of functional coupling properties. **(B)** Depolarization enhances the trafficking of L-type Ca^2+^ channel subunits to the plasma membrane. This key biological process entails the movement of vesicles from the Golgi apparatus to caveolae strategically positioned on the plasma membrane. Abbreviations: AKAP5, A-kinase anchoring protein -5; PKA, Protein kinase A; PKC, Protein kinase C; MLCK, Myosin light chain kinase; MLCP, Myosin light chain phosphatase.

Vascular L-type Ca^2+^ channels are comprised of a Ca_V_1.2 α1 pore-forming subunit along with an auxiliary α2 (150 kDa), δ (17–25 kDa), β (50–78 kDa), and γ (32 kDa) subunit to ensure proper gating, regulation, and trafficking (Catterall et al., 2003; Feng et al., 2018). The Ca_V_1.2 α_1_ subunit retains the transmembrane sequences that confer voltage-gating, and a C-terminus is notable for a diverse array of regulatory sites. L-type Ca^2+^ channels are the primary drivers of Ca^2+^ influx in vascular smooth muscle, and traditional physiology assumes their activity is nearly exclusively set by voltage, with each channel operating independently of one another. Observations collected over the past decade have begun to challenge this dogma by noting that subpopulations of closely situated L-type Ca^2+^ channels work cooperatively with one another to enhance their open probability. First described in 2005 by Navedo and others, L-type Ca^2+^ channels were rationalized to cluster on the plasma membrane in a configuration where the open probability of each individual channel was markedly higher (Navedo et al., 2005). The clustering of active channels creates regions on the plasma membrane of persistent Ca^2+^ influx, resulting from the generation of so-called Ca^2+^ sparklets (Figure 2A). Note, Ca^2+^ sparklets differ from Ca^2+^ sparks, events driven by ryanodine channels on the sarcoplasmic reticulum, as their duration is longer, their amplitude coupled to voltage, and their pharmacological profile distinct (Navedo et al., 2005). The latter is exemplified by the nifedipine block of Ca^2+^ sparklets, akin to that of L-type Ca^2+^ currents; in contrast, nifedipine has no effects on Ca^2+^ sparks. While data is limited, Amberg and others argued that ∼50% of SMC Ca^2+^ current is sparklets-related (Amberg et al., 2007), the remaining current being assigned to non-coupled independent channels.

The regulatory elements driving Ca^2+^ sparklets activity have become a source of active experimental inquiry. Initial work argued that PKCα was an essential activator of Ca^2+^ sparklets, consistent with its impact on the whole cell L-type current (Wesselman et al., 2001; Korzick et al., 2004; Jarajapu et al., 2005; Amberg et al., 2007). Aligning with and building upon these findings, immunohistochemical and TIRF microscopy observations revealed close spatial membrane localization of Ca^2+^ sparklets, Ca_V_1.2 clusters, and PKCα (Santana et al., 2008). The anchoring of PKCα in close proximity to Ca_V_1.2 is achieved through A-kinase anchoring protein, AKAP5, also known as AKAP150 (mice) and AKAP79 (humans), being prominently expressed (Santana et al., 2009; Perino et al., 2012) and capable of binding to the C-terminus of the α1 subunit (Fallon et al., 2009; Dixon et al., 2015). PKCα release leads to displacement of Calmodulin from IQ domain which decreases Ca^2+^-induced inactivation of L-type Ca^2+^ channels (Faux and Scott, 1997; Santana et al., 2009). In light of AKAP5 bringing L-type Ca^2+^ channels in close apposition to PKCα (Coghlan et al., 1995; Oliveria et al., 2007), it follows that Ca^2+^ sparklets regulatory control is lost in AKAP150 deletion mice (Navedo et al., 2008). Subsequent studies have revealed that PKA and Calcineurin also bind AKAP5 in close apposition to L-type Ca^2+^ channels adding another regulatory layer to Ca^2+^ sparklets activity. PKA mobilization leads to phosphorylation of α1 subunit C-terminus at the Ser 1928, increasing the open probability of L-type Ca^2+^ channels (Nystoriak et al., 2017; Prada et al., 2019; Syed et al., 2019; Prada et al., 2020). In contrast, Calcineurin’s effects oppose PKCα, with its activation limiting Ca^2+^ sparklets activity (Navedo et al., 2006; Santana et al., 2009). This Yin-Yang relationship between PKA, Calcineurin, and PKCα can be functionally viewed as creating a flexible platform for Ca^2+^ sparklets regulation (Navedo et al., 2006; Santana et al., 2009).

Moving beyond cellular observations, the next logical question centers on the physiological and pathobiological role of Ca^2+^ sparklets. As to the former, experiments performed on AKAP^−/−^ and PKCα^−/−^ have observed that limited Ca^2+^ sparklets activity coincides with a marked reduction in myogenic tone, suggestive of the former driving the latter (Navedo et al., 2008; Navedo et al., 2010a). Secondly, it has been argued that clustered L-type Ca^2+^ channels are loosely coupled with ryanodine receptors and SERCA pump, thus impacting the Ca^2+^ load/release of the sarcoplasmic reticulum (Navedo et al., 2010b). This idea aligns with: 1) structural data showing close apposition of Ca_V_1.2 clusters with sarcoplasmic reticulum release/uptake proteins; and 2) functional data noting that regions of Ca_V_1.2-Ca^2+^ sparklet activity overlap with areas notable for the tranisent Ca^2+^ release from ryanodine receptors. Moreover, experimental data demonstrates that reducing Ca^2+^ sparklets activity notably slows Ca^2+^ refilling of the sarcoplasmic reticulum (Essin et al., 2007; Takeda et al., 2011).

Considering the preceding physiological observations, it follows that pathobiological processes impinging on Ca^2+^ sparklet activity will be destined to impact the contractile state of vascular smooth muscle. For example, the marked upregulation/AKAP binding of PKCα (hypertension) and PKA (hyperglycemia and diabetes type II) is notable for enhancing arterial tone (Navedo et al., 2008; Navedo et al., 2010a; Navedo et al., 2010b). The latter observation highlights the importance of localized PKA signaling as global activation will relax vascular smooth muscle through hyperpolarization (Navedo et al., 2010a; Morotti et al., 2017; Nystoriak et al., 2017; Prada et al., 2019). Genetic mutations to the Ca_V_1.2 C-terminus, mimicking those observed with Timothy Syndrome, also impact functional coupling and vessel contractility (Navedo et al., 2010b; Napolitano et al., 2014; Priori et al., 2018; Han et al., 2019). So albeit in physiology or pathobiology, the dynamic balancing of PKCα, PKA, and Calcineurin activity is critical to the functional tuning of L-type Ca^2+^ channels as they respond to defined voltage stimuli. The recent review by Dixon and colleagues provides a more detailed examination of this phenomenon. (Dixon et al., 2022).

## Ca2+ channel trafficking and its implication on smooth muscle contraction

An alternative means to enhance the Ca^2+^ influx response to depolarization is to increase the number of L-type Ca^2+^ channels embedded in the plasma membrane. Protein trafficking is key, and work in expression systems provides foundational knowledge of how Ca_V_1.2 subunits are chaperoned to and inserted into the plasma membrane (Figure 2B). Following synthesis in the sarcoplasmic reticulum, Ca_V_1.2 subunits are packaged into vesicle structures which are then guided to the membrane along structural filaments, including actin fibers and microtubules (Simms et al., 2012). This movement is enabled by the key motor proteins, kinesin and dynein, and interestingly these vesicles can switch from actin to microtubules and visa versa, making the trafficking flexible and sensitive to changes in cytoskeletal reorganization (Ross et al., 2008; Smyth et al., 2010). Observing trafficking behavior in tsA-201 cells, Ghosh and others intriguingly noted that a resident pool of Ca_V_1.2 containing vesicles displays a distinct pattern of movement and interaction with the plasma membrane (Ghosh et al., 2018). This included vesicular structures undergoing a dynamic fusion and fission on a second-time scale, with fusion processes displaying “kiss and stay” and “kiss and run” behavior. This study also noted that recently incorporated vesicles displayed Ca^2+^ sparklets activity, consistent with channel clustering, and this process was dependent on the cytoskeleton (Ghosh et al., 2018). While observations are limited in vascular smooth muscle cells, evidence points to caveolae being a site of convergence for L-type Ca^2+^ channels in resistance arteries (Martinsen et al., 2014; Suzuki et al., 2013). Little is known of the stimuli that foster L-type Ca^2+^ channel trafficking, but their targeted transport to caveolae suggests a potential regulatory role for mechanical forces like pressure. This perspective aligns with findings from acute hypertension models, where increased β and α_2_δ subunit expression (Herlitze et al., 2003; Klugbauer et al., 2003) is associated with enhanced surface expression of Ca_V_1.2 subunits and vasoconstrictor drive (Bannister et al., 2012).

## Conclusion and future directions

This mini-review summarizes current thinking on how the functional tuning of L-type Ca^2+^ channels could be tuned in vascular smooth muscle to impact Ca^2+^ influx and tissue contractility. Mechanisms of note include; 1) cooperative gating among neighboring L-type Ca^2+^ channels; and 2) stimulus-induced protein trafficking. Experimental research now defines how each mechanism is regulated by protein kinases, anchoring proteins, cytoskeletal structures, and initiating stimuli. Translating this knowledge into relevant biological settings is the next Frontier and one destined to intrigue the next generation of vascular biologists. Are, for example, the number and size of L-type Ca^2+^ channel clusters truly tuned in temporally concert with the changing physiological environment? Likewise, how do L-type Ca^2+^ channel clusters change in pathobiological settings like sepsis, where the proinflammatory environment progressively leads to circulatory collapse? This deeper understanding of L-type Ca^2+^ channels and its linkage to vascular tone is expected to reveal new conceptual avenues for therapeutic development.

## Abbreviations

AKAP5, A-kinase anchoring protein-5; CPI-17, cytosolic phosphatase inhibitory protein of 17-kDa; MYPT1, Myosin light chain phosphatase targeting subunit 1; PKA, Protein kinase A; PKC, Protein Kinase C; PP1c, Type 1 protein phosphatase 1c.
